# Sex Equitable Prehospital Stroke Triage Using Symptom Severity and Teleconsultation

**DOI:** 10.3389/fneur.2021.765296

**Published:** 2021-11-29

**Authors:** Elin Wiebert, Annika Berglund, Christina Sjöstrand, Einar E. Eriksson, Michael V. Mazya

**Affiliations:** ^1^Department of Clinical Neuroscience, Karolinska Institutet, Stockholm, Sweden; ^2^Department of Neurology, Karolinska University Hospital, Stockholm, Sweden; ^3^Department of Neurology, Danderyd Hospital, Stockholm, Sweden

**Keywords:** acute ischemic stroke, thrombectomy, triage, telemedicine, sex characteristics

## Abstract

**Objectives:** We aimed to determine whether there are sex differences in prehospital accuracy of the Stockholm Stroke Triage System (SSTS) to predict large artery occlusion (LAO) stroke, and endovascular thrombectomy (EVT), and whether clinical characteristics differ between men and women undergoing “code stroke” ambulance transport.

**Materials and Methods:** This prospective observational study collected data between October 2017 and October 2018. We included 2,905 patients, transported as “code stroke,” by nurse-staffed ground ambulance, to a Stockholm Region hospital. Exclusion criteria were private or helicopter transport, onset outside Stockholm, and in-hospital stroke. We compared overall accuracy, sensitivity, specificity, positive and negative predictive values, and clinical characteristics between sexes.

**Results:** No significant sex differences in SSTS predictive performance for LAO or EVT were found, overall accuracy for LAO 87.3% in women vs. 86.7% in men. Women were median 4 years older and more frequently had stroke mimics (46.2 vs. 41.8%). Women more commonly had decreased level of consciousness (14.0 vs. 10.2%) and moderate-to-severe motor symptoms (by 2.7–3.8 percentage points), and less commonly limb ataxia (7.2 vs. 9.7%).

**Conclusions:** The SSTS had equal predictive performance for LAO and EVT among men and women, despite minor sex differences in the clinical characteristics in patients undergoing ambulance transport for suspected stroke.

## Introduction

In 2015, clinical trials established the superiority of endovascular thrombectomy (EVT) over medical treatment in large artery occlusion (LAO) stroke (1). However, EVT is only available at certain hospitals and its benefits diminish rapidly with time, accentuating the need for an accurate prehospital triage.

The Stockholm Region in Sweden has a 2.3 million population, across 6,519 km^2^. The region is served by one comprehensive stroke center (CSC), Karolinska University Hospital, and six primary stroke centers (PSC). Intravenous thrombolysis (IVT) and stroke unit care are provided at all stroke centers, while EVT is only available at the CSC.

Before October 10, 2017, guidelines in Stockholm-mandated code stroke, priority 1 ambulance transport for patients with positive modified face-arm-speech-time (FAST) test or other cause for stroke suspicion raised by the ambulance nurse, presented within 6 h of onset, to the most proximal stroke unit. Patients presenting beyond 6 h of onset, or with unknown time of onset, were also transported with code stroke if displaying critically affected vital signs. On hospital arrival, patients were examined using plain CT and, in eligible patients, IVT was administered. Thereafter, CT angiography (CTA) was performed, unless contraindicated by local hospital guidelines or on clinical grounds. Reasons to abstain from CTA included minor symptoms with >8 h since the last known well, demarcated CT infarct findings fully matching the clinical presentation, contraindications to EVT, such as modified Rankin Scale (mRS) four to five or prestroke life expectancy below 3 months, and contraindications to CTA, such as severe renal failure or contrast allergy. This routine remained unchanged after the Stockholm Stroke Triage System (SSTS) implementation in 2017. When anterior or posterior circulation LAO was found on CTA at the PSC, a CSC stroke physician, with access to electronically transferred images, was teleconsulted on the patient eligibility for transfer for EVT. Reasons for declining transfer were infarct changes > one-third of the middle cerebral artery or greater than one-half of another territory on plain CT, prestroke mRS score 4–5, prestroke life expectancy < 3 months, or CTA results or medical history indicating severe catheter access difficulties. However, age and time since onset or last known well, did not alone affect transfer decisions. Regional quality registry data showed that onset-to-puncture time was 1–2 h longer in secondary transfers, which constituted 75% of the patients treated with EVT, compared with patients directly transported to the CSC when it was most geographically proximal to their place of symptom onset.

The SSTS combines a test for moderate-to-severe hemiparesis with ambulance-hospital teleconsultation, to identify patients with a high likelihood of LAO with EVT indication, for PSC bypass. The SSTS predicts LAO and EVT with high accuracy both within 6 and 6–24 h from onset or last known well, and has reduced regional median onset-to-puncture time in EVT by 69 min, without delaying IVT (2, 3). Studies on AIS have found that, unlike greater mortality, the lower poststroke functional level and quality of life in women remains significant after adjustment for prestroke mRS and age (4–6). To better understand these disparities, research has focused on sex differences in clinical characteristics in stroke. Among patients treated with IVT, women more often have anterior circulation LAO and strokes of greater severity, more frequently caused by cardioembolism because of the atrial fibrillation (7). Traditional stroke symptoms, including hemiparesis, are more common in men compared with women, and the opposite for non-traditional symptoms, such as mental status changes, loss of consciousness, and generalized weakness (7, 8). Presenting with non-traditional symptoms might be associated with a greater risk of misdiagnosis (9). Similarly, the greater frequency of stroke mimics in women could add to differences in diagnostic accuracy (7). It is unknown how any sex differences in the clinical characteristics might affect prehospital LAO triage accuracy.

We aimed to determine whether SSTS triage accuracy for LAO and EVT differs between sexes, and if there are sex differences in the clinical characteristics among patients undergoing code stroke ambulance transport.

## Materials and Methods

This observational study used data prospectively collected between October 2017 and October 2018, in patients undergoing code stroke ground ambulance transportation to a Stockholm Region hospital. Exclusion criteria were private or helicopter transportation, onset outside the Stockholm Region, and in-hospital stroke. Of 2,909 eligible patients, four opted out from the study participation, leaving 2,905 patients in the dataset.

Research Ethics Committee approval was obtained (approval 2017/374) with waived need for active consent. Patients were informed in writing of their right to decline study data collection.

On October 10, 2017, the novel SSTS, using a three-step algorithm, was implemented across the region. In step 1, stroke suspicion was raised by an ambulance nurse, due to positive modified FAST test or other clinical reasons. In step 2, stroke suspects were prehospitally assessed using an NIH Stroke Scale (NIHSS)-derived test for moderate-to-severe hemiparesis, defined as a score of ≥2 NIHSS points each in the ipsilateral arm and leg (the A2L2 test). Test status was classified as positive or negative. The test was inapplicable in the patients presenting with seizures, unconsciousness or bilateral paresis. In step 3, a CSC stroke physician was consulted by telephone on A2L2 positive cases, to discuss the diagnostic suspicion and assess EVT eligibility. This was followed by the destination decision, frequently directly to the CSC, bypassing any more proximal PSC(s). The region-wide electronic health record system allowed CSC physicians to assess prestroke mRS scores of the patients and comorbidities. In A2L2 negative or A2L2 inapplicable cases, a stroke physician at the most proximal hospital (most frequently a PSC) was prenotified of subsequent transportation of the patients there.

**Figure 1:**
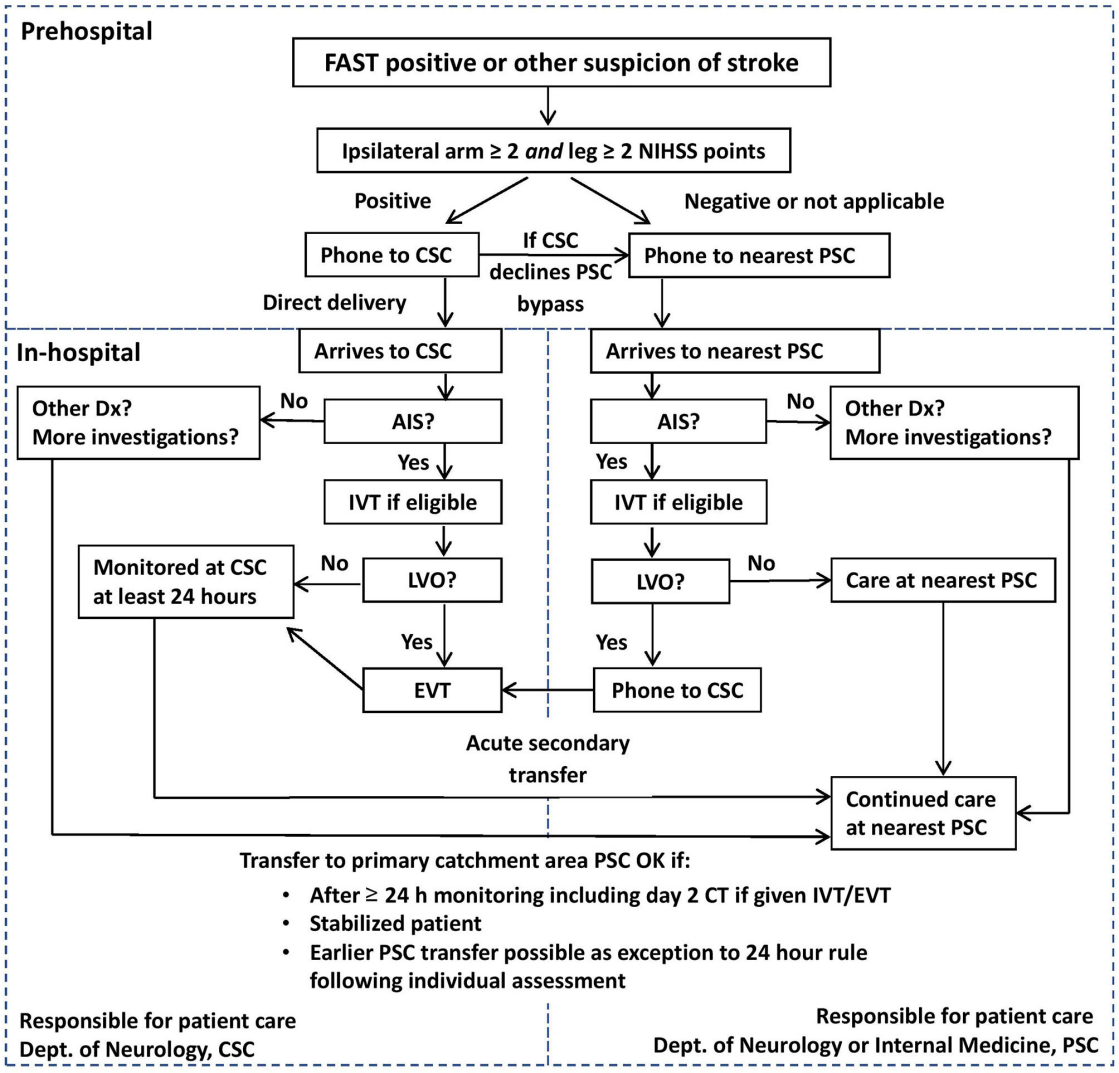
Flowchart of the Stockholm Stroke Triage System.

The SSTS guideline and protocol, including a patient flow chart (Figure 1), was published on the Stockholm Healthcare Region website, and programmed into ambulance portable tablet computers. All the regional ambulances have a crew of two: at least one specialist ambulance nurse (3-year university degree and 1 year of prehospital or anesthesia training) and an ambulance technician (nursing high school diploma and professional training). Prior to implementation, ambulance nurses had web-based training and live lectures, and hospital staff underwent group training.

Triage positive status was defined as suspected stroke with positive A2L2 test and acceptance for direct transport to the CSC following teleconsultation between an ambulance nurse and a hospital physician. Patients testing A2L2 negative or inapplicable, and those testing A2L2 positive but declined PSC bypass because of the EVT contraindications, were classified as triage negative. Reasons for A2L2 positive patients to be declined PSC bypass were low suspicion of stroke, prestroke mRS 4–5, prestroke life expectancy < 3 months and critically affected vital signs requiring stabilization at the nearest hospital. The same classification principles were applied when the CSC was the most proximal hospital, defining triage positivity as confirmed stroke suspicion, A2L2 positivity and absence of contraindications to EVT. LAO stroke was defined as acute ischemic stroke (AIS) with CTA-confirmed occlusion or subocclusion in the arterial segments routinely treated at the CSC: ICA, M1-2, A1-2, P1, BA, and intracranial VA. In the patients with contraindications to CTA, LAO stroke was defined as a dense cerebral artery on plain CT. For analyses of triage accuracy, patients with AIS not undergoing CTA were pooled with patients with CTA confirmed non-LAO AIS. Diagnostic imaging was routinely evaluated by two radiologists. EVT was defined as arterial puncture. Final diagnoses, including mimic diagnoses, as well as stroke and TIA diagnoses, were established during in-hospital care and were obtained from the final discharge notes signed by the senior consultant-level physician responsible for the in-hospital care period.

Triage accuracy measures were sensitivity, specificity, positive and negative predictive values (PPV, NPV), and overall accuracy. Clinical characteristics included sex, age, A2L2 and triage status, use of CTA, site of occlusion or thrombosis, NIHSS total score and subitem scores, final diagnosis, onset-to-needle time (ONT) in IVT and onset-to-puncture time (OPT) in EVT. Pearson's chi-squared test and Mann-Whitney *U*-test were used for comparative analyses of categorical and continuous variables. Two-sided *P* values of < 0.05 were considered statistically significant. All the analyses were conducted using IBM SPSS version 27 (IBM Corp., Armonk, NY, USA).

## Results

Among 2,905 code stroke ambulance-transported patients, 1,420 (48.9%) were women. Clinical characteristics are presented in Table 1. Women were older than men, median 78 vs. 74 years. There were no significant sex differences in triage positive status (10.9 vs. 11.3%) or A2L2 positive test (19.0 vs. 17.5%). LAO stroke diagnosis was made in 10.8 and 11.0% in women and men, respectively. Women more frequently received a stroke mimic diagnosis, 46.2 vs. 41.8%. CTA was less frequently used in women, 44.1 vs. 48.0%. While the overall distribution of occlusion sites showed a statistically significant difference between the sexes (Table 2), the absolute differences were modest, e.g., MCA M1 occlusion in 11.7% of women vs. 8.1% of men. Table 3 shows nearly identical performance of the SSTS in both the sexes, regarding the prediction of LAO and EVT. Overall accuracy for LAO was 87.3 vs. 86.7%, and for EVT 90.7 vs. 90.4% in women and men respectively, with similar nonsignificant magnitudes of difference in sensitivity, specificity, NPVs, and PPVs.

**Table 1:** Clinical characteristics in men and women.

	**Men** **(*****n*** **= 1,485)**		**Women** **(*****n*** **= 1,420)**		***P***
**Characteristic**	**No./total**	**Median (IQR)** **or %**	**No./total**	**Median (IQR)** **or %**	
Age	1,485/1,485	74 (64–81)	1,420/1,420	78 (66–86)	<0.001
A2L2 positive	260/1,485	17.5	270/1,420	19.0	0.294
Triage positive	168/1,485	11.3	155/1,420	10.9	0.733
CTA performed	712/1,484	48.0	626/1,419	44.1	0.037
NIHSS total	1,298/1,485	4 (1–9)	1,221/1,420	4 (1–10.5)	0.007
NIHSS items (>0p^†^)					
1a. LOC	123/1,207	10.2	158/1,127	14.0	0.005
1b. LOC questions	422/1,201	35.1	433/1,122	38.6	0.085
1c. LOC commands	180/1,194	15.1	220/1,116	19.7	0.003
2. Gaze	157/1,194	13.1	168/1,110	15.1	0.171
3. Visual	171/1,187	14.4	173/1,099	15.7	0.372
4. Facial palsy	420/1,196	35.1	410/1,116	36.7	0.417
5a. Arm, left, ≥2 p	165/1,202	13.7	196/1,120	17.5	0.012
5b. Arm, right, ≥2 p	144/1,201	12.0	164/1,119	14.7	0.059
6a. Leg, left, ≥2 p	177/1,200	14.8	207/1,124	18.4	0.017
6b. Leg, right, ≥2 p	160/1,200	13.3	188/1,118	16.8	0.019
7. Limb ataxia	114/1,179	9.7	79/1,093	7.2	0.037
8. Sensory	289/1,191	24.3	283/1,098	25.8	0.405
9. Best language	388/1,199	32.4	375/1,113	33.7	0.496
10. Dysarthria	447/1,195	37.4	399/1,110	35.9	0.467
11. Extinction or inattention	119/1,174	10.1	137/1,091	12.6	0.069
**Diagnosis**					
AIS with LAO	163/1,485	11.0	153/1,420	10.8	0.861
AIS without LAO	295/1,485	19.9	229/1,420	16.1	0.009
AIS, LAO unknown	226/1,485	15.2	231/1,420	16.3	0.438
Intracranial hemorrhage	181/1,485	12.2	151/1,420	10.6	0.188
Stroke mimic	620/1,485	41.8	656/1,420	46.2	0.016
**Treatment**					
EVT	64/1,485	4.3	55/1,420	3.9	0.553
IVT	179/1,485	12.1	158/1,420	11.1	0.435
OPT, min (EVT)	64/1,485	285 (121–373)	55/1,420	299 (134–325)	0.378
ONT, min (IVT)	176/1,485	122 (81–167)	156/1,420	124 (81–160)	0.847

*A2L2 indicates ≥ 2 National Institutes of Health Stroke Scale points each in both the ipsilateral extremities; AIS, acute ischemic stroke; CTA, CT angiography; IQR, interquartile range; IVT, IV thrombolysis; LAO, large artery occlusion; LOC, level of consciousness; NIHSS, NIH Stroke Scale; No., number; ONT, Onset-to-Needle (thrombolysis) Time; OPT, Onset-to-arterial puncture time; p, points*.

† *All NIHSS items categorized as positive if scoring > 0 points, except motor items 5 and 6, which were categorized as ≥ 2 vs. 0–1 points*.

**Table 2:** Site of occlusion or thrombosis.

	**Men** **(*****n*** **= 1,485)**		**Women** **(*****n*** **= 1,420)**		***P***
	**No./total**	**%**	**No./total**	**%**	
**Occlusion site** ^**‡**^					0.001
ICA, extradural only	12/718	1.7	5/640	0.8	0.141
ICA-T	11/718	1.5	17/640	2.7	0.146
ICA+MCA	11/718	1.5	5/640	0.8	0.201
M1	58/718	8.1	75/640	11.7	0.024
M2	47/718	6.5	43/640	6.7	0.898
M3 or more distal	5/718	0.7	5/640	0.8	0.855
ACA	2/718	0.3	4/640	0.6	0.337
PCA	8/718	1.1	13/640	2.0	0.172
Basilar	10/718	1.4	3/640	0.5	0.081
Vertebral	16/718	2.2	4/640	0.6	0.014
No occlusion or thrombosis	538/718	74.9	466/640	72.8	0.375

‡ *On CTA, or, in patients with contraindications to CTA, corresponding to the dense vessel on CT*.

**Table 3:** Triage accuracy for prediction of LAO and EVT.

**Outcome**	**Men** **(*****n*** **= 1,485)**		**Women** **(*****n*** **= 1,420)**		***P***
	**No./total**.	**% (95% CI)**	**No./total**	**% (95% CI)**	
**LAO diagnosis**					
Sensitivity	67/163	41.1 (33.6–48.7)	64/153	41.8 (34.0–49.6)	0.896
Specificity	1,221/1,322	92.4 (90.9–93.8)	1,176/1,267	92.8 (91.4–94.2)	0.657
PPV	67/168	39.9 (32.5–47.3)	64/155	41.3 (33.5–49.0)	0.797
NPV	1,221/1,317	92.7 (91.3–94.1)	1,176/1,265	93.0 (91.6–94.4)	0.803
Overall accuracy	1,288/1,485	86.7 (85.0–88.5)	1,240/1,420	87.3 (85.6–89.1)	0.636
**EVT treatment**					
Sensitivity	45/63	70.3 (59.1–81.5)	39/55	70.9 (58.9–82.9)	0.943
Specificity	1,298/1,421	91.3 (89.9–92.8)	1,249/1,365	91.5 (90.0–93.0)	0.882
PPV	45/168	26.8 (20.1–33.5)	39/155	25.2 (18.3–32.0)	0.740
NPV	1,298/1,317	98.6 (97.9–99.2)	1,249/1,265	98.7 (98.1–99.4)	0.696
Overall accuracy	1,343/1,485	90.4 (88.9–91.9)	1,288/1,420	90.7 (89.2–92.2)	0.806

*CI indicates confidence interval; EVT, endovascular thrombectomy; LAO, large artery occlusion; NPV, negative predictive value; PPV, positive predictive value*.

Out of 2,905 cases, 2,519 (86.7%) cases with available hospital NIHSS scores, the median total was 4 points in both the sexes (Table 1). Regarding NIHSS subitems (Table 1), women somewhat more commonly had decreased level of consciousness (14.0 vs. 10.2%), affected ability to follow commands (19.7 vs. 15.1%), and more moderate-to-severe extremity motor deficits, reaching statistical significance in three out of four extremities (left arm 17.5 vs. 13.7%; left leg 18.4 vs. 14.8%; and right leg 16.8 vs. 13.3%). Conversely, women had a slightly lower frequency of limb ataxia, 7.2 vs. 9.7%.

## Discussion

We found no sex differences in the prehospital accuracy of the SSTS to predict LAO stroke or EVT. While numerous prehospital LAO stroke triage algorithms have been published (10), we have found no publications on sex differences in this setting. Our results are consistent with a study of stroke triage in an urban, academic Emergency Department, and a study evaluating sex differences in a hub-and-spoke hospital telemedicine system (11, 12). Meanwhile, a study in the two US counties in 2005-07 showed somewhat lower sensitivity for prehospital stroke recognition in women (13).

The near-identical performance of our severity- and teleconsultation-based triage system in both the sexes is consistent with the similarity between men and women regarding hemiparalysis, a symptom strongly associated with the presence of LAO stroke (2, 14). Although statistically significant, sex differences in the clinical characteristics among prehospital code stroke patients were minor. We found a somewhat higher frequency of decreased level of consciousness in women upon hospital arrival. This could potentially have affected prehospital triage precision in women negatively, as loss of consciousness has been described as a non-traditional stroke symptom associated with risk of misdiagnosis (7, 9). Meanwhile, a minor such effect could have been offset by slightly more common moderate-to-severe extremity weakness in women, a traditional stroke symptom potentially facilitating correct stroke identification.

Our finding of more stroke mimics in women with suspected stroke is consistent with previous studies (7, 13, 15). This, however, did not apparently detract from the triage performance of the system for LAO stroke or EVT. Our finding of lower use of CTA in women deserves mention. Previous sex difference studies of imaging investigations in stroke have also shown a certain disadvantage for women in the crude analysis, which, however, was no longer evident after adjustment for the higher average age in women at stroke onset (7). The association between age and prestroke functional level could, in part, explain this, as premorbid mRS score 4–5 might in some cases have been considered a contraindication to CTA (since it may have been perceived by the managing physician as not leading to any change of care irrespective of findings) (16). Considering the association between age and comorbidity, and the greater frequency of hypertension among women with AIS, a lower CTA eligibility in women might be caused by a somewhat greater frequency of severe renal failure (17). Unfortunately, our data lack the desired granularity to pursue this question further. Furthermore, with a higher proportion of mimics in women, some of these mimicking conditions will have been diagnosed immediately upon arrival at the Emergency Department, and in such cases, no CTA would have been ordered. Furthermore, some mimics would have been diagnosed on an initial plain CT (e.g., intracerebral tumors), with the radiologist actively choosing to abstain from an arterial-phase CTA, and instead do a non-arterial phase contrast-enhanced CT to better characterize the apparent mass lesion. We are presently conducting further analyses focused on stroke mimics in the SSTS aiming to present those findings in a subsequent publication. Regarding time from onset to thrombolysis and onset to thrombectomy, there were minor differences between the sexes, not reaching statistical significance. While detailed investigation of neuroimaging utilization and reperfusion treatments across sexes was outside the scope of this project, modern studies of sex differences in acute vessel imaging and thrombectomy are warranted, adjusting for prestroke functional level and comorbidity.

Limitations include risk of confounding by age and uncollected variables. The NIHSS was not performed in 386 patients, largely due to lack of perceived clinical indication in patients deemed on hospital arrival to have obvious mimics. It is possible that some of the 457 patients not undergoing CTA due to hospital guidelines, did have LAO stroke, and might have been eligible for EVT in healthcare systems employing other treatment criteria.

## Conclusions

The SSTS had equal predictive performance for LAO stroke and EVT among men and women, despite minor sex differences in clinical characteristics in the patients undergoing ambulance transport for the suspected stroke.

## Data Availability Statement

The raw data supporting the conclusions of this article will be made available by the authors, without undue reservation.

## Ethics Statement

The studies involving human participants were reviewed and approved by Stockholm Ethics Board. Written informed consent for participation was not required for this study in accordance with the national legislation and the institutional requirements.

## Author Contributions

AB and MM contributed to the preparation of the dataset. MM, CS, and EE designed the study. MM supervised the project. EW performed the statistical analysis and wrote the first draft of the manuscript. All authors contributed to manuscript revision, read, and approved the submitted version.

## Funding

MM and CS received funding from the Innovation Fund of the Region Stockholm.

## Conflict of Interest

The authors declare that the research was conducted in the absence of any commercial or financial relationships that could be construed as a potential conflict of interest.

## Publisher's Note

All claims expressed in this article are solely those of the authors and do not necessarily represent those of their affiliated organizations, or those of the publisher, the editors and the reviewers. Any product that may be evaluated in this article, or claim that may be made by its manufacturer, is not guaranteed or endorsed by the publisher.
